# Associations between carcass weight uniformity and production measures on farm and at slaughter in commercial broiler flocks

**DOI:** 10.3382/ps/pez252

**Published:** 2019-05-28

**Authors:** Guro Vasdal, Erik Georg Granquist, Eystein Skjerve, Ingrid C de Jong, Charlotte Berg, Virginie Michel, Randi Oppermann Moe

**Affiliations:** 1 Animalia - Norwegian Meat and Poultry Research Centre, Lorenveien 38, 0515 Oslo, Norway; 2 Norwegian University of Life Sciences, Faculty of Veterinary Medicine, Department of Production Animal Clinical Sciences, PO Box 8146 dep., 0033 Oslo, Norway; 3 Wageningen University and Research, Wageningen Livestock Research, PO Box 338, 6700 AH Wageningen, The Netherlands; 4 Swedish University of Agricultural Sciences, Department of Animal Environment and Health, PO Box 234, 532 23 Skara, Sweden; 5 French agency for food environmental and occupational health safety - Anses Niort 60, rue de Pied de Fond, CS 28440 79024 Niort Cédex, France

**Keywords:** chicken, indicator, poultry, welfare, health

## Abstract

In poultry flocks, flock weight uniformity is often defined as the percent individuals within 10% of the mean body weight (BW) and the variability of this uniformity can be expressed as the CV of BW. Flock weight uniformity is a standardized and objective measured, and could potentially be used as a welfare indicator; however, little is known about the relationship between flock uniformity and other production measures on-farm or at slaughter. The aim of this study was to investigate the associations between carcass weight uniformity (CV of BW) and production measures on-farm and at slaughter in Norwegian commercial broiler flocks. A total of 45 randomly selected mixed-sex Ross 308 broiler flocks were visited prior to slaughter at 28 to 30 D of age (average slaughter age 30.6 D). All flocks were raised under similar farm management systems. The Welfare Quality protocol for broilers was used to assess different animal welfare indicators in each flock. All production data from the slaughterhouse were collected for each flock, including carcass weight uniformity (%), mortality (%), growth rate (g), feed conversion ratio (FCR), and rejected birds (%) in different rejection categories. Univariable and multivariable linear regression models were used to investigate the associations between flock weight uniformity and production and welfare measures. The results showed that flock uniformity varied from 11% to 18% between flocks within the same hybrid, similar management standards, and similar slaughter age (day 29 to 32). Poorer uniformity (i.e., high CV) was associated with increased first week mortality (*P* < 0.004, *r* = 1.48, increased total mortality (*P* < 0.013, *r* = 0.01), increased FCR (i.e., less efficient growth) (*P* < 0.024, *r* = 0.06), reduced growth rate (*P* < 0.0012, *r* = −0.01), and a reduced rejection rate at slaughter (*P* < 0.006, *r* = −0.01). The results show that flock uniformity varies across broiler flocks, and is associated with several production measures.

## INTRODUCTION

Animal welfare in commercial poultry flocks can be monitored using registrations routinely recorded at the slaughterhouse (EFSA, 2012). In broiler production, footpad dermatitis (**FPD**) is now included as a welfare indicator in the European Broiler Directive (2007/43/*EC)*, and should be regularly collected for each slaughtered flock (Ekstrand et al., 1998a; Butterworth et al., 2015). However, animal welfare is considered a multidimensional concept consisting of 3 equally important dimensions: biological function, natural living, and subjective experience (Fraser et al., 1997; Botreau et al., 2007); and although FPD can be considered a useful indicator, it does not provide a comprehensive or complete view of flock welfare (Allain et al., 2009; de Jong et al., 2014). To gain a more complete picture of the welfare situation in broiler flocks, it is therefore necessary to consider several welfare indicators. Slaughterhouse measures can be preferred over on-farm measures in live birds, as these are often less time consuming, recorded on a regular basis, they can partly be automated, and decrease the biosecurity risks inherently related to frequent on-farm visits.

Flock weight uniformity is often defined as the percent individuals within 10% of the mean body weight and the variability of this uniformity can be expressed as the CV of the individual body weights (**BW**). Flock weight uniformity can be used as a measure of how uniform the flock is with regards to body weight during rearing and lay (broiler breeders) (Zuidhof et al., 2015) or at slaughter (broilers) (Feddes et al, 2002). A uniform flock is generally identified with a low CV of BW (usually below 10%) (Feddes et al., 2002; Toudic 2007). Poor flock uniformity (i.e., a high CV) may indicate reduced animal welfare, due to either general housing or management problems, or bird health problems. Poor uniformity may also imply that some birds had trouble accessing feed and water due to, e.g., lameness or disease, resulting in prolonged hunger and thirst in these animals (Weeks et al., 2000; Butterworth et al., 2002). Feddes et al. (2002) reported a negative effect of reduced bird stocking density on uniformity, where broilers housed at 11.9 birds/m^2^ had poorer uniformity (15.3%) compared to higher (23.8 birds/m^2^) stocking densities (13.0%). They suggested this result might be due to the greater floor space allowed the fast-growing birds to grow to their potential. However, compared to broiler breeders, there is relatively limited scientific information on the determinants of flock uniformity in broilers (de Jong et al., 2014). Most publications are related to feed deficiencies Corzo et al, 2004; Gous, 2018). Griffin et al. (2005) reported poorer uniformity in 42-day-old broiler males (CV = 14.2%) compared to broiler females (CV = 12.8%). Furthermore, due to the average faster growth rate of male broilers, there is generally poorer uniformity in mixed-sexed flocks (Gous, 2018). For broilers, poor uniformity is considered negative in an economic context, as the slaughter house requires uniform flocks with the desired mean body weight to meet demands from retailers (Toudic, 2007; Madsen and Pedersen, 2010).

Body weights are measured automatically on the slaughter line worldwide and flock uniformity can therefore be considered a highly standardized and objective measure, recorded routinely. However, little is known about the relationship between flock uniformity and other animal health and welfare measures on farm or at the slaughter house (i.e., the specificity). The specificity of a measure can be defined as the degree of which the indicator is related to a single welfare consequence (high specificity), or to several different consequences (low specificity) (EFSA, 2012). Therefore, as a first step in investigating the potential of using flock uniformity as an animal welfare indicator in commercial broiler production, the aim of this study was to investigate the associations between flock weight uniformity and production measures on-farm and at slaughter in commercial broiler flocks.

## MATERIAL AND METHODS

### Study Design

A total of 45 broiler flocks on 45 different Norwegian broiler farms were visited between January to March 2015, and the Welfare Quality protocol for broilers (WQ protocol) (Welfare Quality, 2009) was used to assess animal welfare at each farm. Most farms in Norway consist of only 1 house; hence, only 1 flock was assessed on each farm. All farms delivered their birds (mixed-sex Ross 308) to the same slaughterhouse, Nortura Hærland, located in the southeast of Norway. Each flock was visited on-farm by the same observer between production day 28 and 30, as close to slaughter as possible (the average age of broiler at slaughter in Norway is 31 D).

Data from the slaughterhouse included for each flock: live weight (g), total mortality % (birds placed – birds received at slaughter), average growth per day (g), average feed conversion ratio (**FCR**) (kg feed/kg live weight), average carcass weight (g), number of birds in 22 different carcass weight categories (50 g intervals from 650 to 1,700 g), FPD score of 100 birds (0 to 200 points), and percentage of rejected birds in 9 different categories. The rejection categories were: color and smell, yolk sac, heart, liver, ascites, slipped tendon, wounds, small, and dead-on-arrival (DOA) %. Rejection due to fecal contamination or technical injuries was recorded separately.

### Farm Visits

All farms were randomly selected from the slaughter lists of about 150 broiler producers, and contacted a few weeks before the visit. Participation in the study was optional, yet all contacted farms agreed to participate and the sample can hence be considered as truly random. One observer, trained by experienced individuals in the theory and practice of the WQ protocol performed all the farm visits. Each farm visit took about 3 to 4h to complete, allowing up to 2 farm visits per day. During every farm visit, the observer was wearing a dark-blue overall with a hood and see-through plastic socks. All data from the farm visits was recorded on site using specialized software on a personal digital assistant (PDA) (Software on the personal digital assistant designed by H. van den Heuvel Wageningen University and Research, Wageningen Livestock Research).

### Sampling Procedure

The on-farm assessments were performed in accordance with the WQ protocol. Only a brief description is given here, detailed description of the protocol is available in the WQ protocol. The visits started with an initial talk with the farmer, where information such as number of animals originally placed, hatchery, parent flock, house dimensions, type of heating, litter type, feed type, mortality, causes of mortality, and number of culled animals was recorded. Then, the observer continued with the assessment in the animal room according to the protocol. Results on the fear test, lameness, and qualitative behavior assessment (**QBA**) from the same flocks has been reported elsewhere (Vasdal et al., 2018; Granquist et al; Muri et al., 2019).

After placing a black dust card in the house, the observer started with the QBA. After the QBA, the touch test was performed, with the observed squatting down in 21 different locations in the house, recording how many animals were within arm's reach, and how many animals that could be touched. After the touch test, 150 birds from at least 5 different locations in the house were gait scored according to 6 categories, ranging from 0 (normal, dexterous, and agile) to 5 (incapable of walking) (Kestin et al., 1992). Litter quality was assessed at the same locations, classified from 1 (completely dry and flaky) to 5 (sticks to boot once the cap or crust is broken). Then, a total of 100 animals in 5 different locations were scored for plumage cleanliness (scored from 0 (clean) to 3 (feathers very dirty)), FPD (scored from 0 (no lesion) to 4 (severe lesion, large area injured)), and hock burn (scored from 0 (no hock burn) to 4 (severe, dark colored lesion of considerable size). At the end of the visit, the amount of dust visible on the black dust card was scored on a scale from 1 (no dust) to 5 (color not visible). The WQ protocol also includes registrations at the slaughterhouse. However, these were not recorded in this study as we wanted to use the registrations that are routinely collected at the slaughterhouse for further analyses.

### Calculation of Scores

The WQ protocol includes detailed descriptions of how to calculate scores based on each measure. Gait score for each flock was calculated by multiplying all animals with score 0 with 0, all animals with score 1 with 1 and so on for 150 scored animals in each flock: ∑ = ((n0*0) + (n1*1) + (n2*2) + (n3*3) + (n4*4) + (n5*5)). The total flock gait score could theoretically range between 0 (all 150 animals receive score 0) and 750 (all 150 animals receive score 5). Thus, an increased gait score indicates increased lameness.

Footpad dermatitis and hock burn score was calculated by multiplying all animals with score 0 with 0, all animals with score 1 and 2 with 1, and animals with score 3 and 4 with 2: ∑ = ((n0*0) + (n1*1) + (n2*1) + (n3*2) + (n4*2)). The total flock score could theoretically range between 0 (all 100 animals receive score 0) and 200 (all 100 animals receive score 2), which is the same procedure commonly used at the slaughter houses. Cleanliness score was calculated by multiplying all animals with score 0 with 0, all animals with score 1 with 1 and so on for all 100 animals: ∑ = ((n0*0) + (n1*1) + (n2*2) + (n3*3)). The total flock cleanliness score could theoretically range between 0 (all 100 animals receive score 0) and 300 (all 100 animals receive score 3). Thus, an increased cleanliness score indicates dirtier birds.

The fear score from the touch test was calculated in accordance with descriptions in the WQ protocol; an index representing the % birds within 1 m is calculated: I = 100 x (number of birds within arm's reach/theoretical number of birds). The theoretical number of birds is equal to the stocking density (birds per m^2^) multiplied with π/2. The index is turned into a score according to spline functions.

When I ≤ 20 then Score = 24.631 + (8.9944 x I) – (0.32423 x I^2^) + (0.0031378 x I^3^).
When I ≥ 20 then Score = 95.660 + (0.46453 x I) – (0.014127 x I^2^) + (8.7479 x I^3^).

These calculations resulted in a touch test score for each of the 50 flocks. The touch test score could theoretically range from 24.6 (no animals touched) to 100 (all animals that theoretically can be touched, are touched).

### Statistical Methods

The data were collected on a handheld computer on-farm and transferred to an Excel (2013) spreadsheet and further to Stata SE 14 (Stata Corp LP, TX, USA). Inspection of the variables was performed in Stata using graphical tools (box plots, histograms, and scatter diagrams), tabulations, calculations of means, medians, standard errors, and 95% confidence intervals. Flock carcass weight uniformity (CV of BW) was the outcome of the analyses. The CV was calculated as the ratio of the standard deviation (σ) to the mean carcass weight (μ) for each flock.

**Figure 1.**
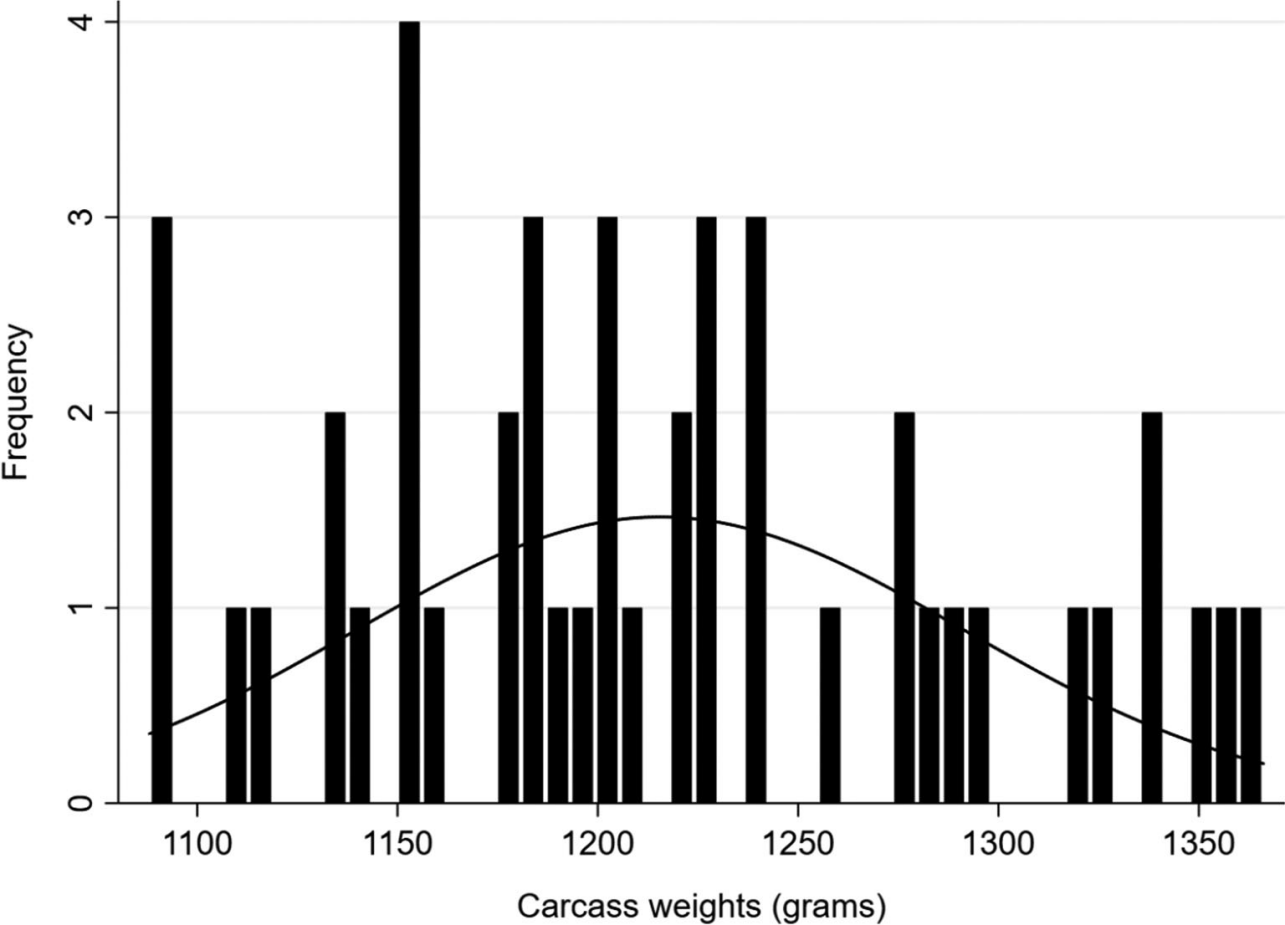
The distribution of carcass weights in the sample population (n = 45). The line represents the normal density plot.

**Figure 2.**
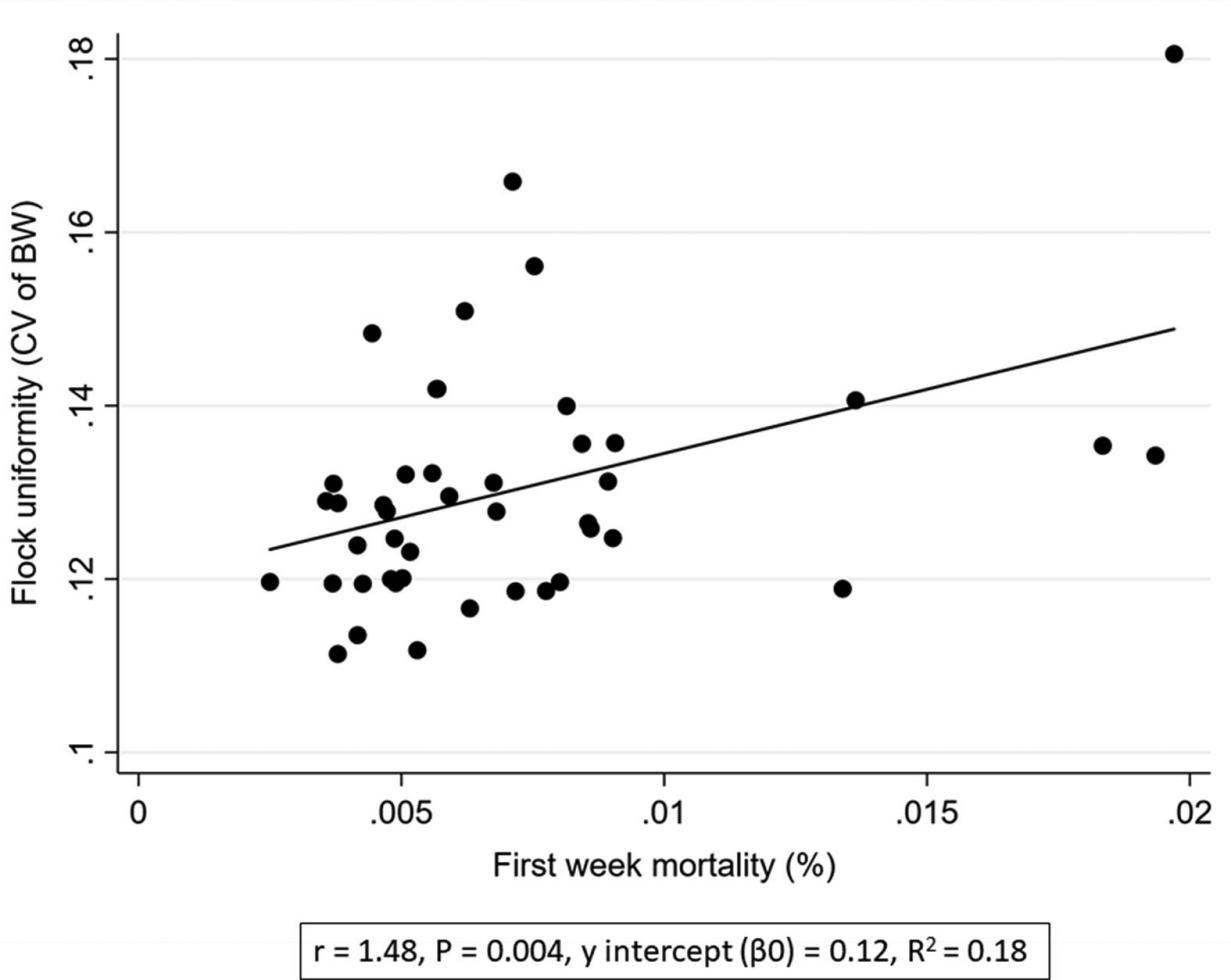
Associations between flock uniformity (CV of BW) (%) and first week mortality (%).

**Figure 3.**
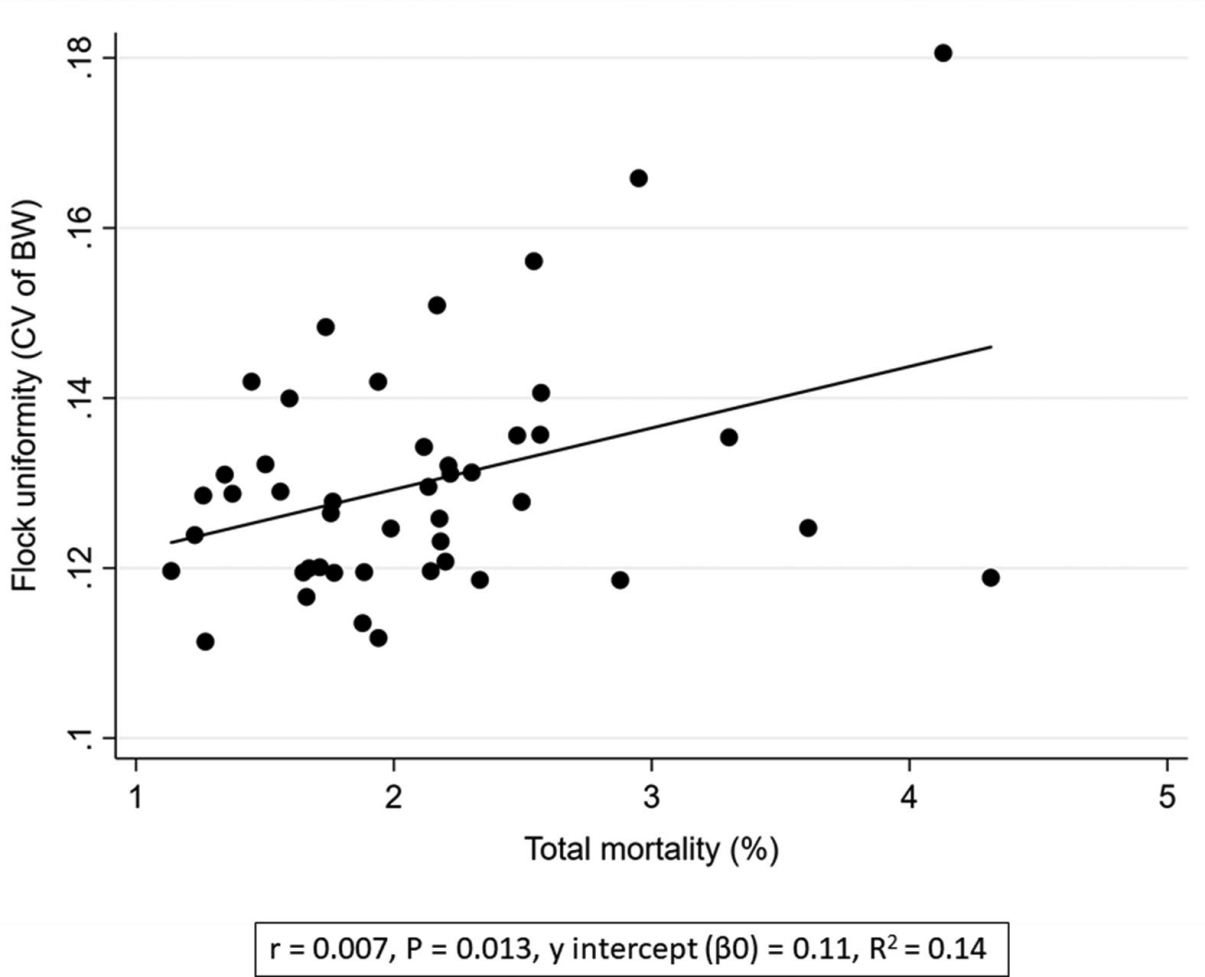
Associations between flock uniformity (CV of BW) (%) and total mortality (%).

**Figure 4.**
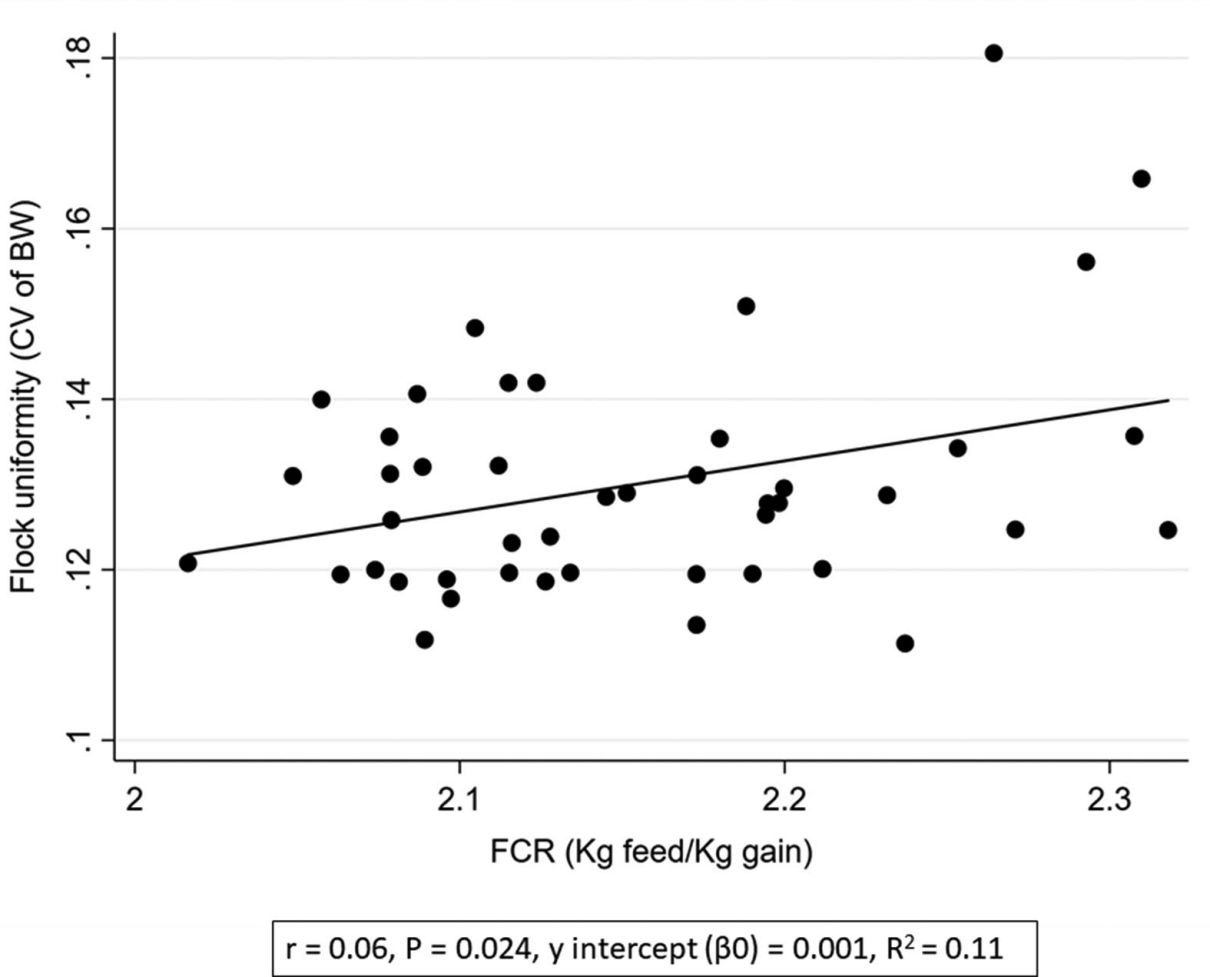
Associations between flock uniformity (CV of BW) (%) and FCR (feed conversion rate; kg feed/kg slaughter weight).

**Figure 5.**
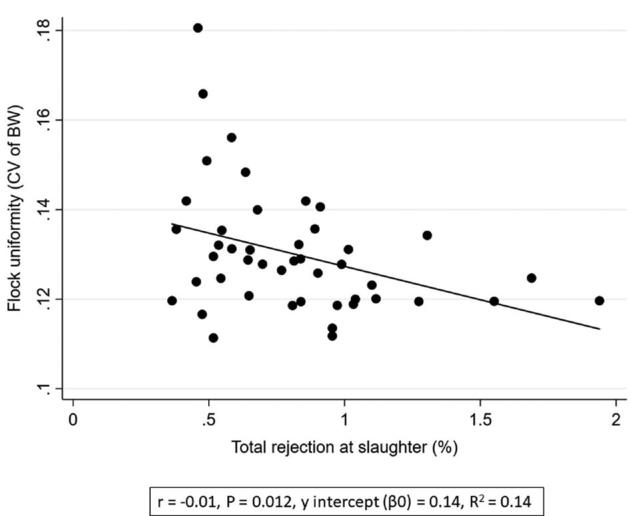
Associations between flock uniformity (CV of BW) (%) and total rejection at slaughter (%).

The outcome variable (CV of BW) was analyzed for associations with any of the independent variables given in Table 1. The outcome variable was approximately normally distributed across the sample population (Figure 1), thus linear univariable regression was used. Residuals were predicted and plotted for normality as shown in Figures 2–5. Associations with *P*-values <0.2 were further analyzed in a multivariable linear regression analyses. A total of 2 models were obtained by backward exclusion until all associations obtained *P* < 0.05. Interactions between independent variables were tested in the final models and were not detected. Residuals were predicted and plotted in normal quantile plots and coefficients of determination (R^2^) were calculated and used to well the model that explains the variability of the response data. The likelihood ratio test was used to observe the improvement of the multiple regression models by inclusion and exclusion of independent variables. Akaike's information criterion and Bayesian information criterion were used to compare maximum likelihood of reduced and full models in which the final models were considered better because of smaller values of the information criterion.

**Table 1.** Overall descriptive production and welfare measures on-farm and at slaughter from the 45 flocks in the study.

Variable	N	Mean	Std. dev.	Min	Max
Slaughter age (days)	45	30.64	0.65	29	32
Number of animals placed	45	17541.18	6389.88	3,900	28,950
Number of animals slaughtered	45	17033.67	6474.74	910	28,472
Flock uniformity (CV %)^1^	45	13	0.1	11	18
Slaughter weight (g)	45	1215.54	75.69	1088.12	1366.11
Growth rate (g/d)	45	39.68	2.12	35.10	44.07
FCR (feed conversion ratio)^2^	45	2.15	0.08	2.02	2.32
Stocking density (kg/m^2^)	45	27.61	3.84	15.54	33.19
Stocking density (animals/m^2^)	45	17.89	2.01	9.14	20.55
Litter quality (1-5)	45	2.28	1.05	1	4.8
Age of parent flock (weeks)	45	36.91	5.96	27	50
Mortality in first week (%)	44	0.01	0.00	0.00	0.02
Total mortality (%)	45	2.11	0.71	1.14	4.32
Culled (% of total mortality)	30	22.94	9.19	8.33	43.46
Dead on arrival (%)	45	0.15	0.12	0.02	0.52
Gait score (flock)^3^	45	260.13	52.83	186	439
Hock lesion score^4^	45	6.22	12.32	0	53
FPD^5^	45	15.51	22.85	0	111
Fear score^6^	45	43.08	30.47	24.63	99.89
Total rejection at slaughter (%)	45	0.82	0.35	0.36	1.94
Color and odor (%)	45	0.02	0.02	0	0.12
Omphalitis (%)	45	0.03	0.04	0	0.27
Heart and circulation (%)	45	0.42	0.45	0	2.97
Liver (%)	45	0.05	0.16	0	1.10
Ascites (%)	45	0.23	0.25	0	1.65
Abnormal growth (%)	45	0.10	0.26	0.00	1.76
Wounds (%)	45	0.05	0.16	0	1.10

^1^Flock uniformity (CV of BW): calculated as the ratio of the standard deviation (σ) to the mean (μ) for each flock.

^2^FCR: Feed conversion ratio (kg feed/kg slaughter weight).

^3^Gait score: 150 scored animals on a 5-point scale/flock: ∑ = ((n0*0) + (n1*1) + (n2*2) + (n3*3) + (n4*4) + (n5*5)), resulting in a flock score between 0 and 750.

^4^Hock burn: 100 scored animals on a 4-point scale/flock: ∑ = ((n0*0) + (n1*1) + (n2*1) + (n3*2) + (n4*2)), resulting in a flock score between 0 and 200.

^5^Footpad dermatitis (FPD): 100 scored animals on a 4-point scale/flock: ∑ = ((n0*0) + (n1*1) + (n2*1) + (n3*2) + (n4*2)), resulting in flock score between 0 and 200.

^6^Fear score: calculated according to the Welfare Quality protocol for broilers: the touch test score could range from 24.6 (no animals touched) to 100 (all animals that theoretically can be touched, are touched).

## RESULTS

### Descriptive Flock Data

The overall descriptive flock data from the 45 flocks in the study are given in Table 1. The flock uniformity varied between flocks, from 11 to 18% (mean 13%, Table 1 and Figure 1). Flock uniformity was not affected by slaughter age, animal density, litter quality, or feed type (Table 2).

**Table 2.** Univariable associations between flock uniformity (CV of BW) and production measures on-farm and at slaughter (n = 45 flocks).

Flock uniformity (CV of BW)	Coefficient (y)	SEM	z	*P* > |z|	95% CI
Litter quality (0 to 5)	0.003	0.002	1.71	0.095	−0.001, 0.007
Peat litter vs. wood shavings	−0.01	0.0072	−1.39	0.171	−0.024, 0.005
Stocking density (kg/m^2^)	−0.000	0.001	−0.60	0.555	−0.001, 0.001
Live weight at visit (g)	−0.000	0.001	−1.84	0.072	−0.000, 0.000
Carcass weight (g)	0.000	0.001	−2.00	0.052	−0.000, 0.000
Growth rate (g/day)	−0.003	0.001	−2.92	0.006	−0.004, −0.001
FCR (feed conversion rate)^1^	0.06	0.03	2.34	0.024	0.008, 0.112
Total mortality (%)	0.007	0.003	2.61	0.013	0.002, 0.013
Feed strength	0.000	0.002	0.23	0.819	−0.003, 0.004
First week mortality (%)	1.478	0.482	3.07	0.004	0.505, 2.451
Culled (% of total mortality)	0.000	0.001	1.10	0.279	−0.000, 0.001
Total rejection (%)	−0.015	0.006	−2.62	0.012	−0.026, −0.003
Abnormal color and odor (%)	0.019	0.086	0.22	0.829	−0.155, 0.192
Omphalitis (%)	−0.480	0.047	−1.02	0.315	−0.143, 0.047
Circulatory disorder (%)	−0.005	0.005	−1.11	0.274	−0.015, 0.004
Liver lesions (%)	0.004	0.013	0.28	0.782	−0.023, 0.030
Ascites (%)	−0.001	0.009	−0.15	0.881	−0.018, 0.016
Abnormal growth (%)	0.005	0.008	0.57	0.575	−0.012, 0.021
Wounds (%)	0.002	0.013	0.14	0.886	−0.025, 0.028
Hock burns (%)	−0.000	0.001	−0.30	0.767	−0.000, 0.000
Footpad dermatitis (%)	−0.000	0.001	−0.76	0.454	−0.000, 0.000

^1^FCR: feed conversion ratio (kg feed/kg slaughter weight).

### Associations Between Flock Uniformity and Production Measures

Increased first week mortality was associated with a poorer flock uniformity (i.e., higher CV) (*P* < 0.004, *r* = 1.48, Table 2 and Figure 2). Poor flock uniformity was associated with increased total mortality (*P* < 0.013, *r* = 0.01, Figure 3). Furthermore, poor uniformity was associated with increased FCR (*P* < 0.024, *r* = 0.06, Figure 4), reduced rejection rate (*P* < 0.012, *r* = −0.01, Figure 5), and reduced growth rate (*P* < 0.006, *r* = −0.01, Table 2). There were no significant associations between flock uniformity and gait score or FPD (Table 2).

The multivariable regression analysis showed that poor flock uniformity was associated with increased mortality (*P* < 0.001, *r* = 0.01) and reduced growth rate (*P* < 0.001, *r* = −0.01, Table 3). A poorer flock uniformity was also associated with to increased first week mortality (*P* < 0.001, *r* = 1.60) and reduced rejection rate (*P* < 0.002, *r* = −0.02, Table 4).

**Table 3.** Multivariable associations between flock uniformity (CV of BW) and production measures on-farm and at slaughter (n = 45 flocks).

Flock uniformity (CV of BW)	Coefficient	Std. error	t	*P* > t	95% CI
Total mortality (%)	0.01	0.001	3.62	0.001	0.004, 0.014
Growth rate (g/d)	−0.00	0.001	3.87	0.000	−0.005, −0.002

Coefficient of determination (R^2^ = 0.36).

**Table 4.** Multivariable associations between flock uniformity (CV of BW) and production measures on-farm and at slaughter (n = 45 flocks).

Flock uniformity (CV of BW)	Coefficient	Std. error	t	*P* > t	95% CI
First week mortality	1.60	0.43	3.68	0.001	0.723, 2.475
Total rejection at PM	−0.02	0.01	−3.35	0.002	0.122, 0.143

Coefficient of determination (R^2^ = 0.36).

## DISCUSSION

The aim of this study was to investigate the associations between carcass weight uniformity (CV of BW) and production measures on-farm and at slaughter in commercial Norwegian broiler flocks. Briefly, the results showed that flock uniformity varied between flocks within the same hybrid, similar management standards, and similar slaughter age. Poorer flock uniformity (i.e., increased CV of BW) was associated with several production measures such as increased first week mortality, increased total mortality, increased FCR, reduced growth rate, and a reduced rejection rate at slaughter, but no associations were found with welfare measures such as gait score or FPD scores.

There is a scarcity of scientific information concerning standard flock uniformity for commercial broiler flocks. Some studies state that a uniform flock is identified with a low CV (usually below 10%) (Feddes et al., 2002; Toudic, 2007), whereas Griffin et al. (2005) reported uniformity in 42-day-old broilers ranging from 14.2% in males to 12.8% in females. Behre and Gous (2008) reported uniformities ranging from 7.8 to 11.8% at 42 D of age in Ross and Cobb broilers housed in metabolism cages to investigate effects of protein content in the feed on uniformity. However, flock uniformity in commercial flocks will likely be larger than in these controlled studies, and the range in uniformity in the present study from 11 to 18% might be as expected. We found that increased first week mortality and higher total mortality during the production period was associated with poorer flock uniformity at slaughter. Mortality during the first week is typically higher compared to the later growth period in broilers (Heier et al., 2002) and first week mortality may be caused by a range of factors. These factors include parent flock age, uniformity and prevalence of diseases, the incubation process, handling at the hatchery, transportation duration, chick quality, and farm management such as care of chick during the placement, hygiene routines at farm, stocking density, feed quality, feeding management, water quality, air temperature, air quality, and season (Heier et al., 2002; Toudic, 2007; Yassin et al., 2009; Kemmett et al., 2014). Furthermore, increased levels of mortality in broiler flocks may be related to a range of factors, e.g., diseases which may also be subclinical in some individuals (Bessei, 2006; Timbermont et al., 2011). Subclinical diseases or other stressors will reduce growth rate in the affected animals, resulting in lower weights compared to the healthy individuals in the flocks, which could explain the poorer uniformity at slaughter.

Another interesting finding is that poorer flock uniformity was associated with increased FCR (i.e., less efficient growth) and reduced growth rate. Both high FCR and a reduced growth rate can be caused by a range of factors such as feed quality, prevalence of diseases, and management (Yassin et al., 2009; Gregersen et al., 2010). All birds in the present study were Ross 308, and hence have similar genetic potential for growth (Havenstein et al., 2003). As decreased uniformity was also associated with increased mortality, the present results suggest that there were 1 or more underlying factors that affected the broilers health and growth rate negatively in these flocks. There were no associations between growth rate and first week mortality, suggesting that a low first week mortality does not necessarily result in a faster growth rate. Biologically, it is well known that male broilers have a faster growth rate compared to females, and weighing around 10% more than females from day 21, resulting in a generally poorer uniformity with increasing age in flock with both sexes present (Griffin et al., 2005). However, all flocks in the present study were mixed-sex flocks of similar age, and any effects of sex would likely be equal in all flocks. Information on uniformity in different sexes at this age has to the authors knowledge not been scientifically presented.

Flocks with poorer uniformity had a reduced rejection rate at slaughter. A possible explanation for this might be that the causes for reduced growth rate and increased mortality may not be rejection causes perse (e.g., subclinical pathologies in the gut or reduced immune status). However, common causes of rejection in broilers include colisepticaemia (Yogaratnam, 1995) and ascites (Olkowski et al., 1996), which are also related to mortality and growth rate on farm. An increased prevalence of these diseases in the flock could be expected to negatively affect the flock uniformity, but there was no association between ascites and uniformity in the present study. The relationship between prevalence of diseases on farm, rejection causes and flock uniformity needs further investigation.

We did not find any associations between flock uniformity and environmental factors such as animal density or litter quality. High animal density has been found to have a negative impact on different broiler welfare issues (Estevez, 2007), including increased lameness (Sanotra et al., 2001) and increased mortality (Dozier et al., 2005), and higher animal densities could potentially be expected to influence flock uniformity. In fact, a previous study found poorer uniformity at lower densities (Feddes et al., 2002), and they suggested that this result might be due to the reduced densities allowed the fast-growing birds in the flock to grow to their potential. The animal densities in the present study (max. 33 kg/m^2^) are lower compared to most European countries, where densities up to 42 kg/m^2^ are allowed (EU Broiler Directive 2007/43/*EC*), and the uniformity in the present study might thus be poorer compared to the rest of the EU. Further studies should investigate the relationship between animal density and uniformity in a larger number of flocks raised under higher densities. Poor litter quality is another well-known factor for broiler welfare, with negative effects on FPD and lameness (de Jong et al., 2014) although we did not find these relationships in the present study. Litter quality could also potentially have a negative effect on flock uniformity, but we found no relationship between litter quality and flock uniformity.

Furthermore, we found no associations between flock uniformity and welfare measures such as FPD or lameness. Kittelsen et al. (2017) found an association between increased first week mortality and increased lameness, and increased lameness could be expected to negatively affect uniformity. Lame birds may experience difficulties reaching resources in the house such as food and water (Weeks et al., 2000; Butterworth et al., 2002; Sanotra et al., 2002), and thus lameness can be negatively related to final weight at slaughter (Gocsik et al., 2014). Furthermore, increased lameness has been associated with a higher mortality in the flock, both through direct associations with infections (Butterworth 1999) or as a consequence of reduced ability to get to the feed and water (Butterworth et al., 2002). Likewise, increased prevalence of FPD in the flock was thought to potentially affect uniformity, either through direct effect on the birds’ feed intake, or through bacterial infections originating from the lesions (de Jong et al., 2014). However, there was a relatively low prevalence of FPD in the present study, making a potential relationship difficult to detect. Further studies are needed to identify whether lameness or footpad dermatitis plays a role in flock uniformity.

The lack of research related to factors affecting uniformity in broiler flocks of similar slaughter age (around 30 D) is interesting. In addition to the potential negative associations with animal welfare, poor uniformity has a negative economic impact on the processor. Furthermore, uniformity may also possibly be a relevant, standardized, and feasible welfare indicator. One of the most widely used welfare indicators in broilers is FPD, which is both a sensitive and a specific indicator for the litter quality in the house (Ekstrand et al., 1997; Ekstrand and Carpenter, 1998b). The sensitivity of an animal-based indicator can be defined as the probability that a given welfare consequence is detected by that measure, whereas specificity can be defined as the degree to which the indicator is related to a single welfare consequence, or to several different consequences (EFSA, 2012). An animal-based indicator with good sensitivity, but low specificity can be used for screening flocks to identify flock with welfare problems, known as “iceberg indicators” or “key indicators” (Kelly et al., 2011). However, we did not find any associations between flock uniformity and welfare measures in this study. Further research on a larger number of flocks is necessary to determine whether flock uniformity may represent a suitable iceberg indicator for welfare problems in commercial broiler flocks.

In conclusion, there were some variations in flock uniformity between the observed flocks, and poorer flock uniformity was associated with increased first week mortality, increased total mortality, increased FCR, reduced growth rate, and a reduced rejection rate at slaughter. The results suggest that one or several underlying factors affected broilers general health status and growth rate in these flocks, resulting in poorer uniformity at slaughter. More studies are needed to investigate the underlying causal relationship between flock uniformity and productions measures reported here, and the potential of including flock uniformity as a welfare indicator in commercial broiler production.
